# Complete Genome Sequence of Klebsiella pneumoniae Myophage Mineola

**DOI:** 10.1128/MRA.00257-19

**Published:** 2019-04-25

**Authors:** Justin X. Boeckman, Lauren Lessor, Jason J. Gill, Mei Liu

**Affiliations:** a Center for Phage Technology, Texas A&M University, College Station, Texas, USA; Georgia Institute of Technology

## Abstract

Klebsiella pneumoniae is an important human pathogen due to the wide range of infections it can cause and its emerging drug resistance. Isolation and characterization of phage infecting K. pneumoniae could be important for future therapeutic applications. Here, we report the complete genome sequence of the T4-like *Klebisella pneumoniae* myophage Mineola.

## Abstract (precis)

Klebsiella pneumoniae is an important human pathogen due to the wide range of infections it can cause and its emerging drug resistance. Isolation and characterization of phage infecting K. pneumoniae could be important for future therapeutic applications.

## ANNOUNCEMENT

Klebsiella pneumoniae is an important opportunistic pathogen due to the continued emergence of highly drug-resistant strains (1), which carry the plasmid-borne and highly mobile K. pneumoniae carbapenemases (*bla*_KPC_) (2). Isolation and characterization of phage infecting K. pneumoniae could be important for future therapeutic applications.

The myophage Mineola was isolated from activated sludge from the municipal wastewater in Bryan, TX, using a plasmid-cured derivative of a KPC-positive (KPC^+^) K. pneumoniae clinical isolate of sequence type 258 as the host. Host bacteria were cultured on tryptic soy broth or agar (Difco) at 37°C with aeration. Phage were isolated and propagated with the soft agar overlay method (3). Phage genomic DNA was prepared using a modified Promega Wizard DNA cleanup kit protocol, as described previously (4). Pooled indexed DNA libraries were prepared with the Illumina TruSeq Nano DNA LT kit, and the sequence was obtained with the Illumina MiSeq platform with the MiSeq v2 500-cycle reagent kit, following the manufacturer’s instructions; this produced 434,532 paired-end reads for the index containing the phage genome. FastQC 0.11.5 (https://www.bioinformatics.babraham.ac.uk/projects/fastqc/), FASTX-Toolkit 0.0.14 (http://hannonlab.cshl.edu/fastx_toolkit/download.html), and SPAdes 3.5.0 (5) were used for read quality control, read trimming, and read assembly, respectively. The genome sequence was closed with PCR with primers (5′-GCCACCCATCATCAAACATATC-3′, 5′-CATCGGGTCGTCGTTCTAAA-3′) to face off the ends of the assembled contig and Sanger sequencing of the resulting product, and the contig sequence was manually corrected to match the resulting Sanger sequencing read. GLIMMER 3.0 (6) and MetaGeneAnnotator 1.0 (7) were used to predict protein-coding genes, which were then manually verified, and tRNA gene prediction was done with ARAGORN 2.36 (8). Putative protein functions were assigned based on sequence homology detected with BLASTp 2.2.28 (9), and conserved domains were detected with InterProScan 5.15-5.40 (10). All analyses were performed with default settings via the Center for Phage Technology (CPT) Galaxy (11) and WebApollo (12) interfaces (https://cpt.tamu.edu).

The phage Mineola genome sequence was assembled into 166,130 bp at 360.5-fold coverage. The genome contains 276 protein-coding genes and 16 tRNAs. Mineola has a GC content of 39.5%, which is 17.65% lower than that of its host (57.14%) (13). It shares 94.1% nucleotide similarity by progressiveMAUVE (version 2.4.0) (14) with *Klebsiella* phage JD18 (GenBank accession number KT239446). Mineola is T4 like, with 204 proteins that share homology with phage T4, as determined by BLASTp (E value ≤ 10^−5^). The main divergences from T4 are in conserved hypothetical genes, which have no homologs in T4 but are conserved among other K. pneumoniae phages, like phage JD18. The Mineola UvsW-like DNA helicase is encoded by two genes; this is a conserved feature among T4-like phages, and the single-gene UvsW in the T4 genome record (GenBank accession number NC_000866) is likely due to a sequencing error (15). All Mineola capsid and tail components have homologs in phase T4 except the putative distal subunit of the long tail fiber, which is 363 residues longer than its T4 gp37 counterpart and is more similar to the T5 L-shaped tail fiber (E value = 4^−103^). A homing endonuclease that shares homology with T4 SegB is embedded in a region containing several tRNAs, which is thought to facilitate spreading of tRNA genes among T4-like phages (16).

### Data availability.

The genome sequence of phage Mineola was deposited under GenBank accession number MH333064. The associated BioProject, SRA, and BioSample accession numbers are PRJNA222858, SRR8788212, and SAMN11259693, respectively.
